# Supplementary material for: Plant-Derived Xanthones against Clostridial Enteric Infections
Source: Antibiotics (Basel). 2023 Jan 21;12(2):232. doi: 10.3390/antibiotics12020232 (PMC9952316; doi:10.3390/antibiotics12020232)
Supplement: Supplementary file 1 [file antibiotics-12-00232-s001.zip › antibiotics-2105925-supplementary.pdf]

Supplementary Materials

Plant-Derived Xanthones against Clostridial Enteric Infections

Ying Liu 1,†, Jianfei Zhu 1,†, Shaoqi Qu 1, Jianzhong Shen 1,2 and Kui Zhu 1,2,*

1 National Key Laboratory of Veterinary Public Health Security, College of Veterinary Medicine, China Agricul-tural University, Beijing 100193, China; liuyingcau@cau.edu.cn (Y.L.); zhujf@cau.edu.cn (J.Z.); qusq@cau.edu.cn (S.Q.); sjz@cau.edu.cn (J.S.)

2 Guangdong Laboratory for Lingnan Modern Agriculture, Guangzhou 510642, China

* Correspondence: zhuk@cau.edu.cn

† These authors contributed equally to this work.

Table S1. Bacteria strains were used in this study.

Bacterial strains	Genotype	Source
S. aureus ATCC 29213		ATCC
S. aureus MRSA T144		*Advanced Science*, **2021**, 8, 2100749
E. faecium CAU369		*Advanced Science*, **2021**, 8, 2100749
E. faecalis CAU544		*Nature communications*, **2022**, 13, 1888
B. velezensis 57-2		This study
CVCC2030	A	CVCC
CVCC60082	B	CVCC
CVCC60101	C	CVCC
CVCC60102	C	CVCC
CVCC60201	D	CVCC
C60-3 82	D	CVCC
E4	E	CVCC
Clinical *C. perfringens* isolates (n = 79)	A	This study

a. ATCC, American Type Culture Collection; CVCC, China Veterinary Culture Collection Center; MRSA, methicillin-resistant *Staphylococcus aureus*; **b.** All 79 *c. perfringens* clinical strains were isolated from ileum contents, cloacal swabs, or feces of chickens, pigs, and cattle from large-scale livestock and poultry farms in the past 3 years and identified and preserved by the National Center for Veterinary Drug Safety Evaluation of China Agricultural University.

Table S2. Antibacterial activities of clinically applied antibiotics against clostridia.

**Clostridia**	**Genotype**	**MIC (μg/mL)**				
		AMG	tylosin	amoxicillin	lincomycin	tilmicosin
CVCC2030	A	0.5	1	≤0.25	2	4
CVCC60082	B	0.5	1	≤0.25	0.5	8
CVCC60101	C	0.5	1	≤0.25	0.5	2
CVCC60102	C	0.5	0.5	≤0.25	0.5	4
CVCC60201	D	0.5	0.5	≤0.25	8	8
E4	E	0.5	1	≤0.25	8	4
19SX 3RK11	A	0.5	1	≤0.25	0.5	8
20SX 1RX187	A	0.5	1	≤0.25	16	8
20SJ BNP12	A	0.5	2	≤0.25	0.5	4
20SJ RX65	A	1	16	≤0.25	32	> 128
20SX 1RX187	A	0.5	1	≤0.25	4	8

Table S3. Antibacterial activities of AMG against 79 clinical *C. perfringens* isolates.

C. perfringens	**Genotype**	**MIC (μg/mL)**	
		AMG	Tylosin
**Standard strains**			
CVCC2030	A	0.5	1
CVCC60082	B	0.5	1
B6261	B	0.5	0.125
CVCC60101	C	0.5	0.5
CVCC60102	C	0.5	0.5
C4989	C	0.5	1
CVCC60201	D	0.5	1
C60-382	D	0.5	0.5
D8346	D	0.5	1
E8084	E	0.5	1
**Clinical isolates**			
21331	A	0.5	1
21343	A	0.5	1
19NM 2CM8	A	0.5	4
19HB BNF	A	0.5	1
19HB BNFC	A	0.5	1
19HB BNFD	A	0.5	1
20SJ BNC14	A	0.5	1
20SJ BNF1	A	0.5	2
20SJ BNG5	A	0.5	1
20SJ BNG7	A	0.5	1
20SJ BNG8	A	0.5	1
20SJ BNP10	A	0.5	2
20SJ BNP12	A	0.5	1
20SJ BNP14	A	0.5	1
20SJ BNP5	A	0.5	1
20SJ BNP6	A	0.5	1
20SJ BNP9	A	0.5	0.5
21SJ BNC10	A	0.5	1
21SJ BNC11	A	0.5	1
21SJ BNC12	A	0.5	2
21SJ BNC13	A	0.5	0.5
21SJ BNC14	A	0.5	4
21SJ BNC4	A	0.5	1
21SJ BNF10	A	0.5	1
21SJ BNF11	A	0.5	1
21SJ BNF12	A	0.5	1

21SJ BNF14	A	0.5	1
21SJ BNG10	A	0.5	4
21SJ BNG4	A	0.5	4
21SJ BNG9	A	0.5	1
21SJ BNP1	A	0.5	32
21SJ BNP12	A	0.5	2
21SJ BNS11	A	0.5	4
21SJ BNS2	A	0.5	2
21SJ BNS5	A	0.5	1
20HB 6RK44	A	0.5	1
20SJ 5RX106	A	0.5	1
20SJ 5RX107	A	0.5	16
20SJ 5RX142	A	0.5	1
20SJ 5RX15	A	0.5	1
20SJ 5RX161	A	0.5	1
20SJ 5RX183	A	0.5	2
20SJ 5RX202	A	0.5	1
20SJ 5RX7	A	0.5	1
20SJ 5RX83	A	0.5	1
20SJ 5RX89	A	0.5	0.5
20SX 1RX10	A	0.5	1
20SX 1RX187	A	0.5	1
20SX 1RX264	A	0.5	2
20SX 1RX284	A	0.5	2
20SX 1RX45	A	0.5	0.5
21HB 5RK19	A	0.5	2
21HB 5RK35	A	0.5	4
21HB 5RK44	A	0.5	4
19SX 3RX70	A	0.5	2
20HB 8PK30	A	0.5	1
20HB 8PK36	A	0.5	1
20SJ 6PK21	A	0.5	2
20SJ 6PK27	A	0.5	2
20SJ 6PK28	A	0.5	1
20SJ 6PK3	A	0.5	1
20SJ 6PK33	A	0.5	4
20SJ 6PK36	A	0.5	4
20SJ 6PK40	A	0.5	1
20SJ 6PKG19	A	0.5	0.5
21HB 2PKG10	A	0.5	1
21HB 2PKG7	A	0.5	2

21HB 2PZ1	A	0.5	2
21HB 3PKG3	A	0.5	32
21HB 3PZ12	A	0.5	2
21HB 3PZ14	A	0.5	4
21HB 3PZ2	A	0.5	4
21HB 3PZ3	A	0.5	4
21HB 3PZ8	A	0.5	2
21HB 3PZ9	A	0.5	32
21HB 5PK8	A	0.5	1
21SX 4PK9	A	0.5	2
21SX 4PKY17	A	0.5	4
21SX 4PKY19	A	0.5	4

Table S4. Antimicrobial activities of AMG in aerobic and anaerobic conditions.

**Organism**	**MIC (μg/mL)**					
	AMG		amoxicillin		tylosin	
	aerobic	anaerobic	aerobic	anaerobic	aerobic	anaerobic
S. aureus ATCC 29213	1	1	0.5	0.5	1	⩽0.25
S. aureus MRSA T144	0.5	0.5	32	32	＞128	＞128
E. faecium CAU369	0.5	0.5	128	128	＞128	＞128
E. faecalis CAU544	0.5	0.5	1	1	＞128	＞128
B. velezensis 57-2	0.5	0.5	⩽0.25	⩽0.25	⩽0.25	0.5

Table S5. Structure-activity relationships for antibiotic activity 10 xanthones.

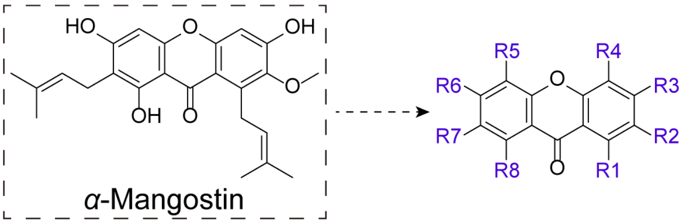

Compounds	Log P	MIC (μg/mL) aginst clostridia				
		A	B	C	D	E
α-Mangostin	4.64	0.5	0.5	0.5	0.5	0.5
β-Mangostin	4.91	1	4	2	2	1
γ-Mangostin	4.38	≤0.25	2	0.5	0.5	0.5
Garciniaxanthone E	5.91	>128	>128	>128	>128	>128
Gartanin	4.38	>128	>128	>128	>128	>128
1,3,7-Trihydroxy-2-prenylxanthone	3.17	16	16	8	16	16
1,4,5,6-Tetrahydroxy-7,8-diprenylxanthone	4.38	>128	>128	>128	>128	>128
1,4,6-Trihydroxy-5-methoxy-7-prenylxanthone	3.05	32	64	16	64	16
3-Isomangostin	4.24	2	2	2	2	2
8-Deoxygartanin	4.77	>128	>128	>128	>128	>128
9-Hydroxycalabaxanthone	3.52	2	4	2	4	4

Table S6. Physiochemical Properties of AMG.

**Phys Chem Profile**		**Lead-like**
MW	410.46	good
log P	4.64	highly lipophilic
H-donors	3	---
H-acceptors	6	good
rotatable bonds	5	good
rings	3	good
globularity	0.069	good
solubility	2.03×10^{-4} mg/L	highly insoluble

Table S7. Stability test of AMG in the gastroenteric fluid.

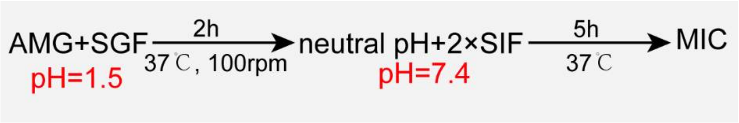

Clostridia	MIC (μg/mL)	
	AMG	AMG+SGF+SIF
CVCC60102	0.5	1

a. SGF: Simulated Gastric Fluid; SIF: Simulated Intestinal Fluid.

Table S8. Primer sequences used in the study.

Primers	Forward/ Reverse	Sequence (5' → 3')
β-actin	Forward sequence	CATTGCTGACAGGATGCAGAAGG
	Reverse sequence	TGCTGGAAGGTGGACAGTGAGG
IL-1β	Forward sequence	TGGACCTTCCAGGATGAGGACA
	Reverse sequence	GTTCATCTCGGAGCCTGTAGTG
IL-6	Forward sequence	TACCACTTCACAAGTCGGAGGC
	Reverse sequence	CTGCAAGTGCATCATCGTTGTTC
Muc2	Forward sequence	ACTGCACATTCTTCAGCTGC
	Reverse sequence	ATTCATGAGGACGGTCTTGG
Claudin	Forward sequence	TGGCCACGTCATGGTATGG
	Reverse sequence	AACGGGTGTGAAAGGGTCATAG
TNF-α	Forward sequence	GGTGCCTATGTCTCAGCCTCTT
	Reverse sequence	GCCATAGAACTGATGAGAGGGAG
ZO-1	Forward sequence	GTTGGTACGGTGCCCTGAAAGA
	Reverse sequence	GCTGACAGGTAGGACAGACGAT

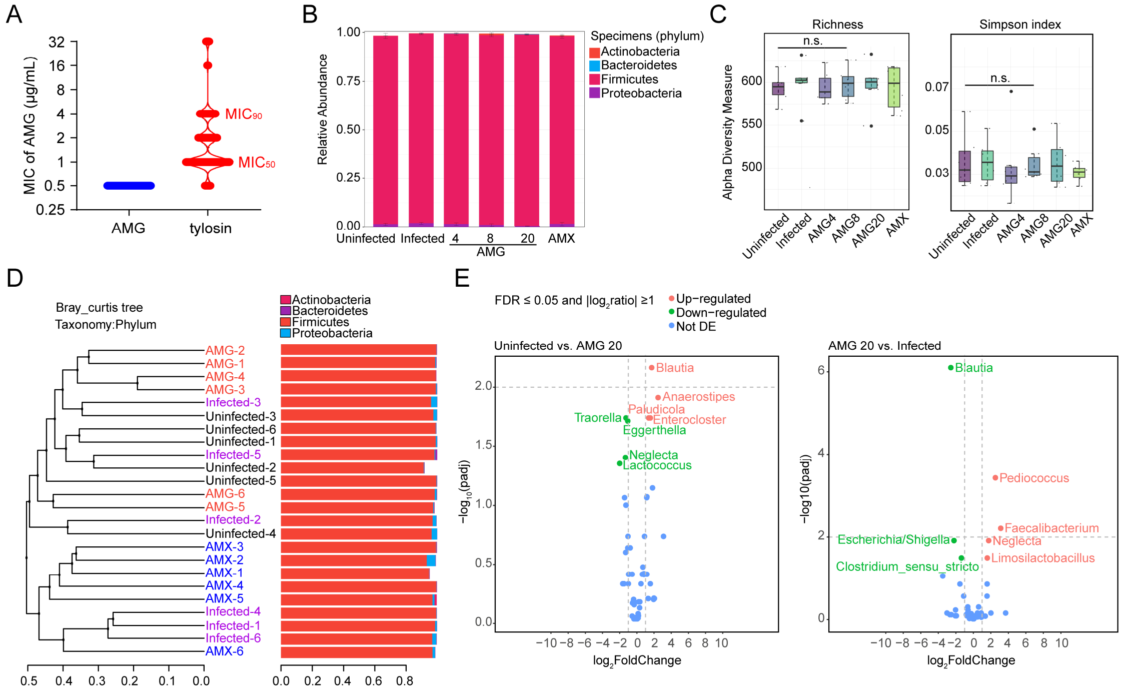

Figure S1. AMG modulates the composition of intestinal microbial community. **(A)** Antibacterial activity of AMG against 79 clinical *C. perfringens* isolates obtained from cattle, chickens, and pigs. MIC_{90} and MIC_{50} values were defined as the lowest concentration of the antibiotic at which 90 and 50% of the isolates were inhibited, respectively. **(B)** Firmicutes were dominant in the cecum of broilers. Relative abundance of different annotated species in cecum microbiota of broilers (Top 4). **(C)** There were no significant changes in species abundance and diversity in the AMG treated group at 4 mg/kg, 8 mg/kg, and 20 mg/kg compared with the uninfected group. Richness and Simpson diversity index calculation of alpha diversity. **(D)** Cluster analysis of the samples showed that the AMG-treated group at 8 mg/kg levels was more similar to the uninfected group. UPGMA clustering tree and phylum level information statistics based on Bray Curtis distance. **(E)** Volcano plots of uninfected group vs. AMG and AMG vs. infected group, comparing the fold change and adjusted *p*-value of microbial species. Vertical dashed lines mark the two-fold change, and horizontal lines characterize the *p*-value cut-off of 0.05.

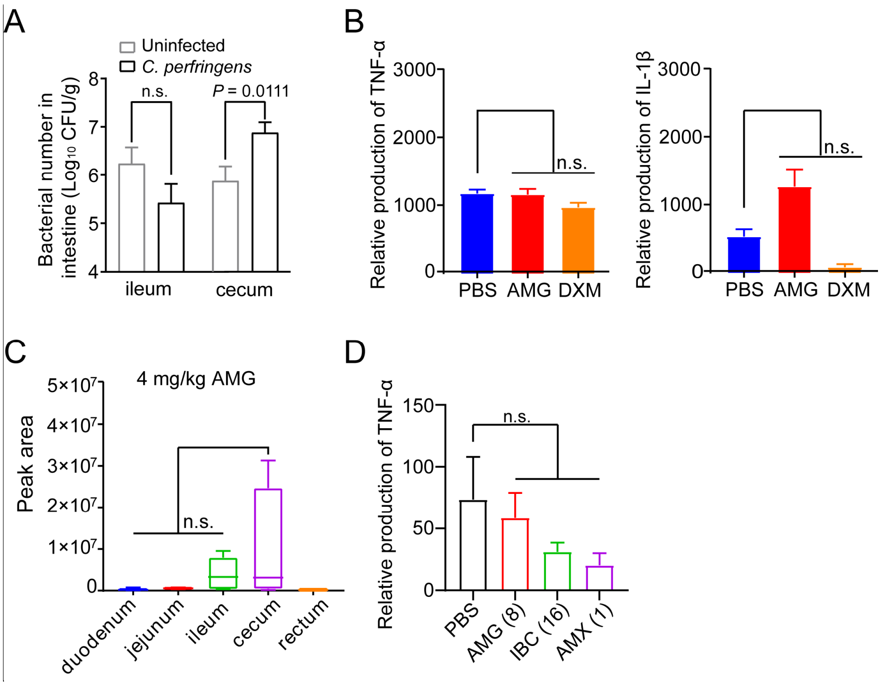

Figure S2. AMG is efficacious in the necrotizing enteritis model of clostridial enteric infections. **(A)** Necrotizing enteritis model was established successfully. The broilers were euthanized after 5 days of persistent infection with *C. perfringens*. Bacterial loads (Log_{10} CFU of *C. perfringens*) in ileum and cecum were counted (n = 6). **(B)** The production of inflammatory factors, including TNF-α and IL-1β, was determined by ELISA. **(C)** Distribution of AMG (4 mg/kg) in different intestinal segments (n = 6). **(D)** Determination of inflammatory factor TNF-α. Chicken TNF-Alpha ELISA Kit was used to measure the content of intestinal TNF-α in chicks of each group (n = 6). All data were presented as means ± SD. *P* values were determined by one-way ANOVA.
